# Understanding the barriers to setting up a healthcare quality improvement process in resource-limited settings: a situational analysis at the Medical Department of Kamuzu Central Hospital in Lilongwe, Malawi

**DOI:** 10.1186/1472-6963-14-1

**Published:** 2014-01-02

**Authors:** Josephine Nana Afrakoma Agyeman-Duah, Antje Theurer, Charles Munthali, Noor Alide, Florian Neuhann

**Affiliations:** 1 Project Officer, Project Fives Alive! Health Directorate, National Catholic Secretariat, P. O. Box KA 9712, Accra, Ghana; 2 Medical Department, Kamuzu Central Hospital, Lilongwe, Malawi; 3 Department of Internal Medicine, St. Katharinen Hospital, Seckbacher Landstrasse 65, 60389, Frankfurt, Germany; 4 Kamuzu Central Hospital, PMB, Lilongwe, Malawi; 5 Institute of Public Health, Ruprecht-Karls University of Heidelberg, INF 324, 69120, Heidelberg, Germany

**Keywords:** Healthcare quality improvement, Patient care, Barriers to quality improvement, Healthcare in resource-limited settings

## Abstract

**Background:**

Knowledge regarding the best approaches to improving the quality of healthcare and their implementation is lacking in many resource-limited settings. The Medical Department of Kamuzu Central Hospital in Malawi set out to improve the quality of care provided to its patients and establish itself as a recognized centre in teaching, operations research and supervision of district hospitals. Efforts in the past to achieve these objectives were short-lived, and largely unsuccessful. Against this background, a situational analysis was performed to aid the Medical Department to define and prioritize its quality improvement activities.

**Methods:**

A mix of quantitative and qualitative methods was applied using checklists for observed practice, review of registers, key informant interviews and structured patient interviews. The mixed methods comprised triangulation by including the perspectives of the clients, healthcare providers from within and outside the department, and the field researcher’s perspectives by means of document review and participatory observation.

**Results:**

Human resource shortages, staff attitudes and shortage of equipment were identified as major constraints to patient care, and the running of the Medical Department. Processes, including documentation in registers and files and communication within and across cadres of staff were also found to be insufficient and thus undermining the effort of staff and management in establishing a sustained high quality culture. Depending on their past experience and knowledge, the stakeholder interviewees revealed different perspectives and expectations of quality healthcare and the intended quality improvement process.

**Conclusions:**

Establishing a quality improvement process in resource-limited settings is an enormous task, considering the host of challenges that these facilities face. The steps towards changing the status quo for improved quality care require critical self-assessment, the willingness to change as well as determined commitment and contributions from clients, staff and management.

## Background

The desire to improve on health systems and healthcare delivery is common in many low and middle income countries (LMIC) [1]. However, the resources required to drive the 'quality agenda’ and the sustainability of quality interventions have encountered major challenges [2]. The requisite knowledge about quality approaches and their implementation are often lacking among health personnel [3]. Therefore, healthcare quality improvement often remains more of a 'verbal expression’, rather than a reality in such settings.

Healthcare in Malawi, as in other developing countries is hampered by chronic lack of resources [4], severe human resource deficiencies [5,6] and inadequate material resources essential for healthcare. At the same time, expectations are high for both the quality of care and training and supervision, particularly in tertiary hospitals [7].

Prior to this study, the management and staff of the Medical Department, Kamuzu Central Hospital in Malawi, where this study was carried out, expressed the concern that their department was not performing up to the expectations of a leading referral hospital in terms of caring for referred patients, teaching of medical interns and clinical officers and conducting supervisory tasks at the district level [8].

In the past, the Medical Department made several attempts to establish itself as a centre of excellence for tertiary care [9], for example with appropriate diagnostic procedures such as gastrointestinal endoscopy and bronchoscopy. However, services such as these were inconsistently available and were dependent on the presence of specific Malawian and expatriate specialists and sufficient nursing staff, among others [10].

Against this background, a situational analysis to identify and understand the systems and the barriers to delivering sustainable high quality healthcare to its clients was performed at the Medical Department. The findings from this study will assist the Medical Department to prioritize potential solutions which will improve the processes required to provide high quality healthcare.

The specific objectives were to assess

1. the organization of patient flow and care within the department

2. the stakeholder views and expectations of the quality of care in the Medical Department

## Methods

### Setting

The Medical Department of the Kamuzu Central Hospital (KCH), is a tertiary referral hospital located in the Central Region of Malawi and serves a population of around 5 million. The hospital is under the direct administration of the Ministry of Health which provides the hospital’s budget. Even though the hospital generates some of its revenue through the paying services, during the time of the study it had no mandate to use the revenue, and instead was required to send such revenues to the Central Treasury of the Ministry of Health.

The Medical Department has a bed capacity of 117 and the entire KCH has over 1000 beds in total. The department has four main sub units, namely the highly dependent unit (HDU), the male and female wards and the medical short stay (MSS) ward. Clinically, the department attends to a host of conditions and cases, both acute and chronic. Outpatient services are provided through two clinics: the main, non-paying OPD unit of the Medical Department (OPD II) and a fee for service paying clinic OPD I. Special clinics for hypertension, diabetes and HIV are run on a weekly basis. The department is also in charge of a small dialysis unit and the TB ward at a different campus, and is further obliged to perform supervisory visits in four districts of the Central Region of Malawi.

### Study approach, sampling and data analysis

A mixed method approach [11] incorporated quantitative and qualitative methods in this descriptive study. Tools for the study comprised of interviews, questionnaires, document review and participatory observation. A triangulated approach for the analysis was reached by including the perspectives of the clients/patients, healthcare providers from within and outside the department and the field researcher. Data collection was carried out in June 2010.

The quantitative aspect of the analysis consisted of conducting structured interviews with 100 outpatients as they exited from the consulting rooms in a convenience sampling approach. Participation in the survey was strictly voluntary. Patients, who were too ill to follow and answer the interview questions properly, were excluded. Only patients and not their families or legal representatives were interviewed.

The exit interview sought to address research objective 1 by identifying:

• the patient's perspective of the quality of healthcare delivery and

• the patient’s satisfaction with services in the Medical Department

### Tools for data collection

Guidelines were developed for the in-depth interviews, and a structured questionnaire was developed by the authors, adapting some of the quantitative elements from the *AJK 2008 Questionnaire*. This validated questionnaire was developed for a quality improvement exercise in a hospital in Pakistan that has a setting similar to that of the KCH. In order to validate the questions before posing them to the study interviewees, the questionnaires for this study were first administered to some staff of the KCH.

### Interviews

All the questionnaires for both in-depth and structured interviews were originally designed in English. The structured outpatient questionnaire was subsequently translated into Chichewa (local Malawian language) for clear communication with interviewees who could not speak or read English. Responses in Chichewa were then translated back into English through an interpreter. Quantitative data from the interviews were analyzed with *Microsoft Excel and Epi-Info Statistical Programme (CDC Atlanta)*.

The qualitative methods included participatory observation and in-depth interviews with key stakeholders. The data collection tools were developed in conjunction with partners in the Medical Department.

For the in-depth interviews, 20 stakeholders were purposively sampled [12] and stratified into four categories of respondents entailing *staff, management, patient and affiliate* (details of this composition can be found in Additional file 1: Appendix 3). The framework for the sampling involved selecting different cadres of care providers and patients in different units of the Medical Department to provide a comprehensive picture of the quality situation in the Medical Department. The qualitative interview sought to address research objectives 1 and 2 by identifying:

• awareness and practice of a culture of quality improvement in the Medical Department

• stakeholder views, perception and expectations of quality of care

• pattern of patient flow and utilization of services within the department

Interviews were audio recorded and later transcribed. Content analysis of the transcribed data was done inductively based on the grounded theory by identifying analytical categories that emerged from the data by following Pope’s five steps of qualitative analysis: familiarization, identification of a thematic framework, indexing, charting, mapping and interpretation [13].

After the initial in-depth interview analysis, there was no need for more interviews, since the emerging themes had reached maximum saturation.

### Document review and observation

The document analysis included a review of outpatient records at the OPD II during the time of the field study. These records provided information on the average patient density, patient flow and pattern of service utilization in the Medical Department, as well as the common diagnoses and presentations to the OPD II. The review was structured to identify referrals to or from the OPD II, but this information could not be determined from the OPD registry.

A qualitative approach of probing (questioning) was used to document processes in the department. The principal researcher served as a participatory observer. By working in the field for a period of one month, she was able to observe firsthand the usual practices within the Medical Department.

### Ethical considerations

The study proposal was reviewed and approved by the research committee of the Institute of Public Health, Ruprecht-Karls University of Heidelberg, Germany and by the National Health Science Research Committee (NHSRC) of Malawi. No monetary or other forms of compensation were given to respondents. Permission was given for respondent views to be tape-recorded. All information received and reviewed was handled with the strictest confidentiality.

## Results

The results reported below reflect the views of the different stakeholders interviewed.

### Structured patient interviews

Out of the 100 sampled outpatients, 98 responded and only two declined. Unless stated otherwise, all analyses were performed on the results from 98 respondents. Overall satisfaction was very high among interviewees: 59% of the respondents rated their satisfaction level as 'very good’, 40% as 'good’ and only 1% as 'poor’.

General satisfaction was further divided into three categories: Out of 95 responses, 45% liked the staff performance, 34% liked the patient focus in the hospital/Medical Department and 16% liked 'other things’.

Three options: *staff performance, patient focus* and *other areas* were provided for respondents to identify what they thought were the major problematic areas or bottlenecks in the department. Out of the 82 respondents answering this question, 41% of them cited 'staff performance’ as the major problem/bottleneck, 27% suggested 'patient focus’ in this regard and 32% thought problems were due to 'other areas’ including 'shortage of drugs’ , 'long waiting times’ , 'whom-you-know service’ (queue jumping) and 'poor amenities in the department’. Table 1 further describes the patient provider relationship that emerged from the structured patient interviews.

**Table 1:** Patient-provider relationship during consultation

**Item**	**Yes (%)**	**No (%)**	**Respondents**
**Privacy given**	99	1	97
**Health conditions told**	88.7	11.3	97
**Treatment implications told**	79.4	20.6	97
**Opportunity to ask questions**	62.8	37.2	78
**Your preferences considered**	91.1	18.9	56
**Told how to take your medication**	95.7	4.3	92
**Treated with respect**	97.9	2.1	97
**Informed on staying healthier**	70.1	29.9	97
**Physical exam performed**	90.6	9.4	96
**Informed to come for check-up**	60.5	39.4	76

### Stakeholder in-depth interviews

The responses by stakeholders from all categories were grouped into three main thematic areas of 'existing quality elements and models’ , 'challenges or problematic areas hindering good quality practices’ and 'recommendations for quality improvement’.

Emerging themes from the in-depth interviews are summarized in Table 2 and grouped under thematic areas: 'existing good quality models in the Medical Department’ , 'weaknesses or challenges of quality improvement’ and 'suggestions for improvement’.

**Table 2:** Overview of themes on quality emerging from in-depth interviews by various stakeholders

**Respondent group**	**Strengths/existing quality model**	**Weaknesses/challenges hindering quality improvement in healthcare delivery**	**Areas for improvement**
**1. Affiliate respondents (n = 5)**	- Existence of treatment protocols	- Staffing- related issues: shortage, underperformance and poor attitude	- Procure essential resources for patient care
	- Quality control and assurance measures are in place	- Scarcity of resources	- Use resources efficiently
	- Improved patient care practicies	- Patient care	- Enhance team work with supporting departments
	(Additional file 1: Appendix 1, Section 1.1)	- Lack of some patients taking responsibility for their own care	(Additional file 1: Appendix 1, Section 1.3)
		(Additional file 1: Appendix 1, Section 1.2)	
**2. Patient respondents (n = 4)**	In-patients described their overall satisfaction of the care they receive as	- Poor amenities and services in the department	- Strengthen staff attitude and performance
	• 'very good’ (1 view)	- Weak adherence to treatment protocol	- Involve patients in their treatment and management
	• 'good’ (2 views)	- Patients not involved in their own care	- Improve amenities in the department
	• 'poor’ (1 view)	(Additional file 1: Appendix 1, Section 2.2)	(Additional file 1: Appendix 1, Section 2.3)
	(Additional file 1: Appendix 1, Section 2.1)		
**3. Staff respondents (n = 4)**	- Staff attitude and performance is good	- Workload	- Provide supervision and training
	- Existence of treatment protocols	- Poor patient care	- Encourage patient-centred care
	- Availability of some logistics like the computer for e-learning	- Lack of adherence to treatment protocol by some staff	- Ensure accountability of and by staff
	(Additional file 1: Appendix 1, Section 3.1)	- Staff-related issues: inadequate training and supportive supervision, low incentives for work	- Encourage effective communication among staff
		(Additional file 1: Appendix 1, Section 3.2)	
**4. Management respondents**	1. Effort to maintain quality	• The medical department is perceived to be the weakest department in KCH for quality of health care delivery(Additional file 1: Appendix 1, Section 4.1)	1. Civil society should be involved in sensitizing patients and holding health staffs accountable to patients
4.1 Current state of quality of healthcare	(Additional file 1: Appendix 1, Section 4.1)	• Inadequate human resource	
		• Lack of some essential diagnostic tools	
		• Limitation in the use of the few available diagnostic tools	
4.2 Patient care and patient focus	1. Patients appreciate staffs when satisfied with service given	1. Patients complaint about wrong prescription or delayed treatment	
		2. Weak patient involvement in their treatment plan	
		3. Self referrals and weak patient referral system among referring facilities	
4.3 Treatment protocols	• Treatment protocols are available and accessible to all staffs	1. Non-compliance due to personal preferences among prescribers; ignorance on the relevance of protocol use; lack of drugs to prescribe	
	• There is a planned review of the current protocols		
4.4 Change management	1. Emergency cases are attended to in the MSS ward before transferring to intensive care	3. No defined human resource plan to cater for staff who leave	
	2. Team system for focused patient care and ward rounds		
4.5 Management-related issues		• Shortage of staff	
		• Lack of training and proper orientation for staff	
		• Poor staff attitude (Additional file 1: Appendix 1, Section 4.5.1)	
		• Weak accountability by staff (Additional file 1: Appendix 1, Section 4.5.2)	
		• Weak leadership structures	
		(Additional file 1: Appendix 1, Section 4.5.3)	
		• KCH as a tertiary hospital wastes resources by attending to many primary level cases	
		(Additional file 1: Appendix 1, Section 4.5.4)	
		• Disintegrated data management system in the hospital	
		• No strategic plan for the hospital (Additional file 1: Appendix 1, Section 4.5.5)	

The patients in this survey – both inpatients and outpatients - considered the quality of care in relation to how patients can receive better attention from care givers and be treated with respect as being very important. In their view, good quality included receiving both medical and non-medical care in a manner they perceive to be good.

The staff respondents identified the lack of proper patient management as one of the Medical Department’s problems. Proper management here refers to care which is safe, effective, patient-centred, timely, efficient and equitable [14].

Findings from the in-depth interviews related to staffing included most prominently the lack of staff [5], and at the same time, certain attitudes of some health workers such as laziness, complacency and absenteeism.

## Document review and participatory observations

### Staffing

The medical staff in the Medical Department from January to December 2010 included two clinical officers (certified physician assistants), seven medical doctors (four expatriates and three Malawians), one nursing manager (matron), 24 nurse midwife technicians and six registered nurses. The support staff comprised 14 cleaners, three auxiliary nurses, three patient attendants and two clerks. There were also interns and students-on-rotation, who were assigned on duty, but their exact numbers were not known and varied frequently. Clinicians work in teams led by a senior clinician, and the teams alternate daily regarding the admission duty. Patients are followed by the same team from admission to discharge. Officially, three nurses are on day shifts and two are on night shifts in each of the three medical wards: 2A (female ward), 2B (male ward) and the Medical Short Stay (MSS) ward. All support staff are on day shifts only.

Poor communication among staff and between staff and patients was reported in the interviews and observed to affect the quality of care in the Medical Department. Such comments included, 'illegible and or incomplete notes in patient files’, 'irregular staff attendance to handing over meetings’ and 'rare updates on the day-to-day management of the hospital from management to staff’ (Additional file 1: Appendix 1, 3.2.4).

### Patient flow and care

**Figure 1:**
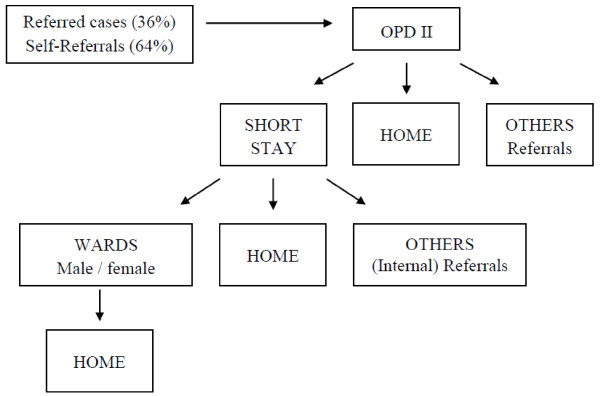
Patient flow pattern at the medical department, June 2010.

Patient flow information, as depicted in Figure 1, is a summary of processes described by the Medical Department staff and confirmed by the researcher’s onsite observation. Throughout the Medical Department, the researcher observed sufficient directional signs which made it easier for patients and visitors to find their way round the department without significant difficulty. For those who could not read, there were security and other service personnel to assist with patient flow.

All admissions are through the MSS where patients are observed, treated and discharged or transferred to the main ward, as required. The MSS ward carries a large proportion of the daily work load for patient management, receiving patients when the general OPD is closed during evenings and weekends.

The patient flow pattern again shows a higher use of the MSS ward during late hours; 41% of the 1077 admissions in June 2010 were during the day shift (8 am to 4 pm) and 59% during the night shift (4 pm to 8 am). The Medical Department OPD II recorded a total of 1015 patients, with 437 males and 578 females in June 2010, which translates to an average daily attendance of 42 patients to the Medical Department during the study month. This data excludes visits on Sundays, since the unit is closed then.

### Registered diagnoses to the OPD II

The OPD diagnosis record for June 2010 showed a large diversity, namely approximately 104 different conditions and cases (see Additional file 1: Appendix 2 for full list), some of which actually refer to the same disease or diagnosis, e.g. general body pain and body ache. The entries in the register do not follow any agreed thesaurus or standard terms. In many cases, ward clerks copy the presumed admission diagnosis from patient charts or books without confirmation by the admitting clinician.

## Discussion

This study identified profound deficiencies in the system functions of patient care, staffing and the management of the Medical Department of KCH. It illustrates that the staff are indeed aware of these problems and open for change. This, in itself, is a prerequisite for the next necessary steps that will include agreeing on achievable goals and defining and implementing “fixes” for quality improvement.

The observations of the selected group of interviewees concurred with the main themes and matched also the researcher’s observations and extractions from registers. Thus, the described results appear to be robust in triangulation.

Findings from this study have been related to the literature on similar studies, carried out to either confirm what is already known or to contrast what other studies may have reported in similar investigations.

### Documentation

The study revealed important shortcomings in documentation in the OPD registers as reported in the results under heading 'registered diagnosis to the OPD II’. These data gaps presuppose that some staff do not appreciate the essence of keeping good registers and maintaining proper data management which is a tool to securing high quality of healthcare delivery.

The findings on improper data management suggest the need for a simplified checklist tool of diseases and conditions for clinicians and clerks to use in order to correctly describe the case load. Furthermore, much attention needs to be paid to the documentation of diagnosis at discharge, since this should provide more solid information on causes for admission and it is crucial for the continuum of care after discharge [15].

### Patient flow, statistics and care

In a tertiary hospital, the WHO recommends that cases seen should require tertiary care [16]. In this survey, however, 64% of the 95 respondents to that question indicated that they are self-referrals to the KCH Medical Department (Figure 1). This observation may indicate poor functioning of the healthcare referral system or the lack of patient trust in the first and secondary levels of care [17]. The implications of a weak referral system on the quality of care constitutes a further strain on the limited resources - both human and material - and the inability of the Medical Department to fully concentrate on its mandate as a tertiary unit in focusing on tertiary care, teaching and research [16]. This also poses a conundrum: The department is being used by patients like a secondary level of care facility and currently the department can offer tertiary level of care only for a few conditions. This finding underlines the need to re-define the role of the department, including its relation to referring centres and hospitals. Furthermore, the defined function needs to be reflected in staffing and equipment.

Central hospitals have frequently been challenged to absorb numerous staff and resources compared to first- and second-line health providers, without delivering the respective quantity and quality of care. If left undefined, the problem will continue and create frustration with patients, staff and the wider health system [18].

The MSS in the Medical Department has been functioning as a triage unit, where critically ill patients are observed and discharged or admitted when necessary. The MSS has worked to reduce the burden on bed occupancy in the main wards [19] while improving on patient outcomes. The unit is thus functioning as a central and crucial point for delivery of quality care within the department: a practice in the Medical Department that is similar to the accident and emergency ward for triaging cases. The statistics on patient flow through the MSS, as recorded in the results, shows higher patient volumes at night than in the day, yet more staff were assigned to the MSS during the day shift than during the night shift. Improving the quality of care delivery, does not therefore always demand new resources, but often the more effective management of the few available resources [14]. Looking at data can assist the management to make informed decisions.

The number of patients followed during the participant/researcher observation was not of much interest to the patient flow patterns, rather the observation of patient flow was to give a broad overview of patient movement within the Medical Department. In contrast, the OPD registers have been used to establish the patient flow density and patterns of services assessed in the department.

Information obtained relating to the quality of patient-provider relationships was found to be encouraging, yet providers are to be encouraged to involve patients more in their own management, by allowing them to ask questions and explain their feelings and or fears about the care they receive.

### Staffing-related issues

The human resource crisis in health is a huge challenge, not only in the KCH Medical Department but a shared problem throughout Malawi [20]. This challenge emanated as a common theme from the stakeholder interviews. In instances where there is a shortage of staff, the few available staff need to be efficiently managed to handle their workload without feeling demotivated by their work demands [6].

A match of the rate of patient flow to the staff capacity, as described in the Results section, (understaffing) would suggest that staff workload is not a problem as strongly indicated by the interviewees. However, it must be stressed that being a teaching hospital, the mandate of the medical team is not only limited to caring for patients, but also to teaching, research and outreach support to the sub districts. It follows that the views of the stakeholder interviewees about inadequate staffing may be a valid point in spite of the data of staff-to-patient ratios in the June 2010 OPD records.

Assuming the OPD data reported in this study is a true reflection of patient density at the Medical Department, then this observation may imply an inefficiency and underutilization, or misallocation or even absenteeism of the staff strength in the unit [21]. On the other hand, if there was considerable underreporting of patients in the registers, then this may be a sign of serious data gaps and thus call for some data quality checks in the Medical Department. Again, if the patient load and the interviewee assertions about staff shortage are anything to go by, then in the attempt to ensure quality improvement in care delivery of the Medical Department, increasing the staff capacity and reducing staff turnover needs to be addressed promptly, and no longer as merely a known but 'neglected’ issue [5]. To address the staffing needs, the leadership of the Medical Department has to provide data to inform management decisions: for example, about patient needs and how to meet the needs with the staff available [22,23]. This argument aligns with Øvretveit’s recommendation of designing a standard for management quality which will guide service efficiency [24].

Communication in healthcare is very crucial for the efficient management of patients. With the observed gaps in both verbal and written communication among staff, the Medical Department could adopt the SBAR (Situation-Background-Assessment-Recommendation) model to enhance communication among staff and to improve patient safety [25].

### Leadership

This study equally confirms Øvretveit’s observation [24] that managers sometimes feel they have little control over health workers. In the Medical Department and KCH at large, management mentioned that it is struggling with little or no accountability from staff, low or lack of commitment and low staff motivation (Additional file 1: Appendix 1, Sections 4.5.1, 4.5.2). Staff and management interviewees attributed the lack of accountability from staff to weak leadership structures (Additional file 1: Appendix 1, 4.5.3) at the departmental level and the centralization of authority over the health workforce at the national level. Therefore, neither the hospital management nor the heads of departments have control over their staff or are able to hold them accountable for their actions, omissions or absenteeism (Additional file 1: Appendix 1, 4.5.2.). According to Kotagal [26] leadership is one of the cornerstones of sustainable quality improvement, as continuous weak leadership structures may further compromise the quality of care delivery.

### Stakeholder view

The quality of healthcare is defined and influenced by people’s experiences, perceptions and expectations [27]. While patients may be more concerned about non-medical procedures like provision of food or cleanliness (Additional file 1: Appendix 2), staff may focus on how to apply evidence-based care to patients. Both perspectives are needed to complement each other for effective quality of care interventions in the Medical Department.

According to the definition of Peach [28] the high rate of patient satisfaction in this survey would imply that a high quality of care exists in the Medical Department (refer to *Results*, Section 4.1). However, relying on indicators like satisfaction alone does not prove that good quality of care exists [29]. It might also mean that patients do not know what to expect. It is, therefore, more helpful if service clients are asked to discuss different components of healthcare and base their judgement on the different aspects rather than to give a broad and general description of such a multifaceted entity like a hospital or a complex interaction like healthcare delivery. It is noteworthy that the interviewed patients do not openly complain about the limited capacity for diagnosis and treatment at the department, as the staff respondents did.

This attitude can partially be explained by social desirability [30], i.e. whether patients do not know about better treatment options or whether they know and simply accept the status quo.

Although the study employed adequate measures to ensure that respondents could speak freely and sincerely during and after the interviews without being victimized by staff, it may be possible that some respondents were apprehensive about getting poorer care if they were forthcoming about any shortcomings in their own care at the Medical Department [31].

Among the providers, quality of care was considered to be good in a hospital if the staff is satisfied with the work they do and patients appreciate the care they receive. Required here is a conducive environment where resources are available, staff work output is recognized through salary or incentives, and an end result of improved health for their patients (Additional file 1: Appendix 2). From the responses, however, the Medical Department is under-resourced to fully execute its duties [2] as a tertiary hospital (Additional file 1: Appendix 1, 1.2).

## Conclusions

In order to reverse the process of staff becoming overwhelmed by their tasks, becoming complacent, demotivated and giving poor performance, it is essential to identify modifiable factors that are under the control of the Medical Department. It is worth noting that planning for quality improvement measures requires patient, staff and management involvement to establish where they stand on the quality ladder. All involved parties need to have achievable goals and define and implement appropriate processes for such. The willingness of staff members to engage in the change process must be recognized as being indispensable. Tasks for tertiary healthcare facilities in resource-limited settings, like the Medical Department of KCH in Malawi, are multiple and constitute a huge challenge for the staff. Quality improvement initiatives can serve as a tool for staff empowerment and better quality of patient care.

## Competing interests

The authors declare that they have no competing interests.

## Authors’ contributions

All authors were involved in the conception and design of the study and have all approved the final version of the manuscript to be published. The corresponding author wrote the first draft, and all authors have been responsible for redrafting and revising the intellectual content of this article.

## Pre-publication history

The pre-publication history for this paper can be accessed here:

http://www.biomedcentral.com/1472-6963/14/1/prepub

## Supplementary Material

Additional file 1 Appendices, Tables and figures. Click here for file
