# Supplementary material for: Understanding the barriers to setting up a healthcare quality improvement process in resource-limited settings: a situational analysis at the Medical Department of Kamuzu Central Hospital in Lilongwe, Malawi
Source: BMC Health Serv Res. 2014 Jan 2;14:1. doi: 10.1186/1472-6963-14-1 (PMC3880175; doi:10.1186/1472-6963-14-1)
Supplement: Additional file 1 — Appendices, Tables and figures. [file 1472-6963-14-1-S1.pdf]

Understanding the Barriers to Setting Up a Healthcare Quality Improvement Process in Resource-limited Settings: A Situational Analysis at the Medical Department of Kamuzu Central Hospital in Lilongwe, Malawi

APPENDIX 1: STAKEHOLDER IN-DEPTH INTERVIEWS

The views of the stakeholder interviewees have been presented according to their defined category in this study (affiliate, in-patient, management or staff) to allow for easy distinction between how different stakeholders in healthcare perceive or define quality and how they would want quality of healthcare to look like in the Medical Department.

1. AFFILIATE VIEWS

Out of the 5 sampled respondents,4 participated in the interview and their views are presented under emerging themes, categorized into three sections: *existing quality models in the Medical Department, problematic areas* and *recommendations* for quality improvement in the Medical Department.

1.1 Existing quality models in the Medical Department: Models of quality identified included *treatment guidelines, quality control and assurance* and *improved patient care.*

Affiliates identified some quality structures and processes that exist or existed in the Medical Department and which have been contributed to better care delivery. '…*We actually set aside a purpose public structure which is the Lighthouse to look after HIV infected and affected people so that took off a lot from the Medical Department so that they concentrate on the other more conventional conditions, …'* ([1]AFF03/30-06-10/49). Respondent AFF04/07-10/50 made mention of some quality assurance measures that were made in the Medical Department between 2007 and 2009 to improve patient care: '…*regular (ward) rounds, triage of patients on admission, regular in-services, development of protocols, establishment of the admission-/short-stay ward, improvement of commitment of staff….'* The supporting units like the laboratory have put in

[1] Coding system where AFF stands for affiliate interviewees, and the format of coding is interviewee identification number/date of interview/age of respondent.

place measures of quality control which will make them better serve the Medical Department with accurate lab results.

1.2 Problematic Areas: The three problematic areas that face the Medical Department according to the affiliate respondents include: *staffing related issues, scarcity of resources* and *patient care.*

Staffing was the commonest problem area raised several times by all respondents. The problem of staffing has to do with shortage of, under performance of and the attitude of some of the 'few' available staff. One respondent refers to the shortage of staff as '*…a very big chronic problem…chronic shortage of staff because as you expand you expect more patients and if the number of providers remains the same it will pose pressure (on the staff)…'*AFF01/29-06-10/58. Describing staff performance in the Medical Department, one respondent made mention of shortage of staff as a probable cause of incompetence on the part of some medical officers who make poor diagnosis and wrong drug prescription for patients, *'… somebody is just, is like copying, editing, then copying and pasting like what the other doctor prescribes…Yes, human resource, that is why I'm saying human resource because maybe they (staff) are not so many; at times there is only one clinician or doctor who is looking at all that queue (of patients) so maybe he is tired …that is why he is doing that copying and pasting…'*AFF03/02-07-10/36.

The attitudes of staff which undermine the quality of care delivery was described by one respondent as due to '*lack of commitment from staff*' (AFF04/07-10/50) and by the other as due to '*…just negligence…'*AFF03/02-07-10/36.

Scarcity of resources like drugs and other medical consumables is one of the major obstacles facing the Medical Department. According to AFF01/29-06-10/58, *'the outcry (from the Medical Department) has been shortage of some of the reagents and supplies.'*

Affiliate respondents identified that patients are not properly managed in the Medical Department especially with clinicians' non-compliance to treatment protocols, '*…they (clinicians) just prescribe any drug anyhow; …now what we see is that they are not following the Malawian treatment guidelines. I don't know how those patients are getting cured …, it is either*

*by the grace of God or…, because the clinician(s) are not sure, of what sort of combination of drugs (to prescribe)…'*AFF03/02-06-36.

Respondent AFF03/02-07-10/36 added that patients are sometimes the cause of the poor care they receive in the Medical Department; '*…that is also another problem from the patient, because if the patients come here… I mean when somebody is so serious, they cannot even talk so even those clinicians/physician they fail knowing where to start (from)'*

1.3 Recommended areas for improvement: The affiliates recommended that for quality of healthcare to improve in the Medical Department, essential resources for patient care should be available; the 'scarce' resources available should be used effectively and team work with supporting departments like the laboratory and pharmacy should be enhanced.

2.0 PATIENT VIEWS

Respondents were from the 2A, 2B and 4B paying wards. Out of the five respondents, three were referred patients and two were self referred. The themes from the in-patient interviews have been categorized similarly to the affiliate views: *existing quality models; problematic areas and recommendations.*

2.1 Existing quality models: The existing quality practices identified by in-patient respondents are expressions of their satisfaction of the services in the department. One patient respondent rated her overall satisfaction with the Medical Department as 'Very good'; two respondents said their overall satisfaction is 'Good', one however said it was 'Bad' and one other did not give any rating. One interviewee commented that: *'Patient care is there always ... they care. The hospital is a bit better.'* [2] IP02/30-06-10/28

2.2 Problematic areas: Areas identified include *staff-patient relationship; amenities and services in the department* and *treatment protocols.*

---

[2] coding system where IP stands for in-patient interviewees, and the format of coding is interviewee identification number/date of interview/age of respondent

Concerns were raised about poor communication between patients and care givers as well as patients not been involved in their own management. IP04/30-06-10/30: '… *they just come and do what they want to do but they don't ask for my consent.* A contrasting view from IP05/02-07-10/30 was '…*yes, always (they ask for my consent)*. IP04/30-06-10/30 narrated, '… *there is no one to explain what is going on..., There was a time I was given a drug and I was feeling itchy and I asked to know what drug I had taken but they told me I can't know the drug, only them (health workers) can know and they provide treatment of care and I couldn't proceed to ask more questions.'*

Interviewees also commented on the amenities and other non-medical services provided in the Medical Department which to them, do not define quality of care: *'...food is provided here but I'm waiting for my wife to bring me food... because they don't bring it (the food) bed by bed. They just call out to people to come for food but as it is, I can't walk for my food',* IP02/30-06-10/28. One interviewee added; *'sometimes it (the ward) smells bad, sometimes, they just clean outside but not under the beds'*IP04/30-06-10/30.

On the treatment protocols, I heard through informal conversations and through observations that, sometimes patients' preferences do not permit care givers to comply with the treatment protocols with reasons being the knowledge gap between patients and care providers about some medical procedures.

2.3 Recommendations: In-patients mentioned the following areas which need to be improved to ensure better quality of care: *staff attitude and performance; involving patients in their treatment and management* and *improving amenities in the department*

*'... I think the nurses should be counselled. They should be patient because they deal with different kinds of people...',*IP04/30-06-10/30.

'*... they just give the drugs and I take them I prefer that the health worker explains the relations of drugs',* IP05/02-07-10/30.

IP05/02-07-10/30: '*I get water to drink from the toilet taps and to me, it is not hygienic.*

I will suggest they have taps in the rooms' (IP04/30-06-10/30).

3.0 STAFF VIEWS

Themes from staff interviews have been categorized under *existing quality models, problematic areas* and *recommendations from staff.*

3.1 Existing quality models: *staff attitude and performance, treatment protocols, facilities in the Medical Department.*

3.1.1 Staff attitude and performance: Staff mentioned their motivation to give off their best in their day to day service in the Medical Department. ST03/29-06-10/21:'*my source of motivation is my colleagues; the interns, senior doctors and the consultants, my seniors; it is like they inspire me ... I admire them, they are my role models and I want to be like them.'* ST02/28-06-10/45 also said, '*I like Medical Department ... we work hand-in-hand'*.

3.1.2 Treatment Protocol: ST03/29-06-10/21 admitted the importance of the protocols as: *'... yes, we were also following them (treatment protocol) every time; because when we have attended to a patient, we are supposed to submit them to a senior colleague. So he asks questions which will demand us to use the protocols to defend ourselves…'*

3.1.3 Facilities: One of the facilities that enhance staff performance was mentioned to be the computers: '*…yes, the computers, they have improved us because now students who do not have laptops can use them to type their presentations. You can also access the internet to get information…*' ST03/29-06-10/21

3.2 Problematic areas: *workload, patient care, treatment protocol and staff-related issues.*

3.2.1 Workload: Respondents complained about the workload in the Medical Department. ST03/29-06-10/21 mentioned, *'... most of the time we have workload,... we should be working with the consultants but most of the time you find us working alone, which is not supposed to be like that… it is like we were made to do work which was not meant for us.'*

3.2.2 Patient care: one interviewee explained that patients do not always receive the best of care due to work because according to him, '… *we are supposed to do a ward round on patients but*

we cannot do that and the patients might be discharged without a check sometimes due to work constraints…', ST03/29-06-10/21.

3.2.3 Treatment protocol: ST03/29-06-10/21 mentioned some challenges staff sometimes face with patients in using the treatment protocols. *'… like when someone needs oxygen he can refuse… perhaps they think we are trying to kill them … some (patients think) that, you are sucking the life out of them. In such instances, 'we stop because when he or she dies the family will hold me responsible.'*

3.2.4 Staff related issues: includes *shortage of staff, low incentives for work done, lack of training; almost absent supportive supervision* and *poor communication among staff.*

ST01/28-06-2010/42: '*…there are no allowances for night duty…'* the interviewee illustrated on communication as: '… *Even when there is a change in director, we are not informed…just here there is a new director, but we are not informed officially…'*

3.3 Recommendations to improve the quality of care

4.0 MANAGEMENT VIEWS

The views of management have been analyzed under five main themes: *the current state of quality of health care in the Medical Department, patient focus component of quality in the department, treatment protocols, change management,* and *management issues in the Medical Department in line with quality of care.* Most of the comments from interviewees were on the challenges management is facing regarding quality of healthcare delivery. The overall perceptions and responses from the management interviewees have been quite negative compared to other categories of interviewees.

4.1 Current state of quality of health care in the Medical Department and KCH: Describing the overall quality in the Medical Department and KCH in general, interviewer MG01/29-06-10/45 said: '*poor, very poor, the rate of quality (in this hospital) is very poor…the Medical Department is the poorest.'* However interviewer MG01/29-06-10/36 was of the opinion*: I will just say it (quality) is above average, it is not hundred percent (in the Medical Department).* MG02/29-06-10/55 commented on how the Medical Department has improved in some ways

'…*when I first came here in 2006, we had no medicines, we only gave them food. But within these four years, I have seen a remarkable improvement in the supplies and some improvement due to NGOs support.'* The challenge of achieving quality in the department was addressed as: '*the biggest problem is human resource. Human resource is responsible for the delay in care, it is responsible for equipment not being available, it will explain everything….'* MG04/02-07-10/41 added that some of the challenges include '*the limitations in making diagnosis of most diseases (and) lack of diagnostic tools'.* He also mentioned about the situation in the wards where some of the patients have to sleep on the floor because sometimes the bed capacity is less than patient inflow.

4.2 Patient care and focus component of quality in the Medical Department: The views of how patient-centred care is embedded in patient management in the Medical Department, how the correct clinical knowledge is applied in every situation and the challenges of proper case management were sought and some of the views expressed are:

MG05/05-07-10/32: '*I think that they (patients) complain that the treatment was not right; sometimes they complain about delay in treatment. The positive things are sometimes just thank you but not anything special.'* According to interviewee MD03/29-06-10/36, patient-centred care is not integrated in the Medical Department, it only comes to play when there is a serious problem between a patient and a staff which needs to be resolved; '*as of now we do not have a component whereby the patients have a direct say or something like that on the quality… patients are involved for instance, if a mistake has happened and may be the patient has complained…unless there is a problem and unless somebody starts a thing, patients are not really involved…'* To institutionalize a patient-centred care as part of quality of care in the Medical Department, interviewee MG01/29-06-10/45 mentioned that; '… *(we need) a third party (civil society) to make sure that it sensitizes the way we handle problems…'*

Describing the pattern of patient referrals, MG05/05-07-10/32 mentioned that, 's*ometimes they (patients) come with letters or something just written out, even sometimes you cannot trace the medical officer (who referred the patient)….'* At the moment, referrals in the Medical Department are not monitored and a lot of times, patients come to this tertiary hospital without first seeking treatment in a primary or secondary health facility. Also two way referrals are not

well organized; from observations and the interviews, treatment details of referred patients are only written in their health passports but no feedback is given to the referring hospital or health centre.

Further on patient care, there is a missed opportunity for staff to learn from past experience to improve patient care in the Medical Department. For example, there is no mortality and morbidity meeting to review causes of death, negligence or any medical error. Again on deaths, there is no system to offer any recognized support to guardians when patients die and so most often in the Medical Department, the bereaved wail so loud that it could be very disturbing to other in-patients and staff.

4.3 Treatment protocols: Management interviewees admitted that although the treatment protocols are useful for patient care, there is a problem of some staff not complying with the protocols. The reasons given were due to ignorance, lack of motivation and lack of the needed drugs to prescribe. MG05/05-07-10/32: *I think there (are) some people who do not know their use and they are being used rarely and quite often not enough…sometimes I think (there is no) motivation to use the tools.* However there is no defined system for monitoring or providing any supportive supervision for staff on the use of the protocols. MG05/05-07-10/32: '*I think it all depends on the team leader; like I try with my team to refer to the protocols, but basically there is no monitoring of staff adherence to the protocols'*,. For the review process of the treatment protocols, the interviewee mentioned that the protocols were introduced only last year but they will be reviewed soon.

4.4 Change management: respondents commented on changes that have influenced positively or negatively on the quality of care delivery in the Medical Department and the challenges of managing changes. MG05/05-07-10/32: '*…we have lost two specialists; so we have no specialist here in the department; the motivation has gone down and there has also been shortage of staff'.* The challenge of change management in the Medical Department according to MD01/29-06-10/45 is '*lack of control over human resources'*. Positive changes mentioned include the setting up of the short stay where patients are reviewed before being admitted to the main wards and the team system of attending to patients. '*… I can say the number one change is on the way the doctors and the clinicians conduct the ward rounds,…they have put themselves in teams … and this system is actually working…'* MD03/29-06-10/36

4.5 Management issues in the Medical Department in line with quality of care delivery: the following concerns came up as some of the direct management-related challenges in quality improvement in healthcare delivery: *Staffing, Accountability, Leadership, Function of the hospital, Data and data management.*

4.5.1 Staffing: management described challenges on staffing as: *shortage of staff; lack of training and proper orientation for staff* and *attitude of staff (such as indiscipline and lack of motivation to work).*

4.5.2 Accountability: It also came up several times that there is little or no accountability of staff to anybody; people do what they like and they are almost never questioned. One respondent also attributed the lack of accountability from staff as due to the hospital not having direct control over its staff: *' that is why we are knocking on the doors of the Ministry of Health that we want reform… to have power over human resource'* MG01/29-06-10/45

4.5.3 Leadership: MG01/29-06-10/45: '…*you don't know who the Head of Department in the Medical Department is; they are fighting there for headship… when the headship is not defined, you want to think that most of the things are not running well, isn't it?'* MG05/05-07-10/32 added, '…*the challenge I think is the leadership; if someone is brought from outside I think he will be taken more seriously…'*

4.5.4 Function of the hospital: '… *you find the specialists are bringing in primary health care services in this hospital. You see they want to go and do primary health care but… we need to cultivate that culture; of thinking that we are a tertiary (hospital) and we need to concentrate on our core functions which are service provision, training and providing support'* MG01/29-06-10/45

4.5.5 Data and data management: Data management as I observed, is a big quality problem in the Medical Department; from the collection, processing and management of data for use in the Medical Department and even in the whole hospital there are so many lapses in data collected and valuable data are left on shelves, in boxes and sometimes lost. Apart from proper documentation and management of data, most essential management data are absent in the Medical Department and hospital in general. For example, there was no definite strategic or

annual plan for the Medical Department or the hospital as at the time of the interviews; *Interviewer*: '*What are some of the strategic plans for the hospital?'* MD01/29-06-10/45: *The most strategic plan for this hospital since it was created will be developing one right now. We have requested yet…for a consultant to help us to develop the strategic plan.'*

Again, data management is disintegrated and therefore there is the lack of a reliable data set for the hospital to use for its decision making: '*the data which is processed at (these different units) do not get to management. ... Management runs this hospital on the basis of I don't know'*

APPENDIX **2: ALL CONDITIONS PRESENTED TO AND RECORDED IN THE OPD II REGISTER IN JUNE 2010**

**The unexplained abbreviations and unclear conditions italicized in the table below reflect the obvious weakness in documentation and poorly defined internal consistency. The data does not distinguish between follow up and new cases such as is observed in the high frequencies in hypertensive and diabetic conditions.*

**Conditions presented**	**Frequency**
1. Abdominal discomfort	1
2. Acute Diarrhoea	1
3. Acute renal failure	2
4. Allergy	1
5. Anaemia	19
6. Angina	1
7. Acute respiratory infection	8
8. Arm pain	1
9. Arthritis	3
10. Ascites	8
11. Asthma	82
12. Back ache	1
13. Brought in dead	8
14. Body itching	1
15. Bronchitis	29
16. Cryptococcal Meningitis	3
17. Candidiasis	1
18. Cardiac effusion	1
19. Congestive Cardiac Failure	10
20. Cryptococal meningitis	3
**Conditions presented**	**Frequency**
21. Chest pain	2
22. Cirrhosis (liver)	1

23. Constipation	3
24. CPT	1
25. Cerebrovascular accident	3
26. Diabetes	215
27. Diarrhoea	6
28. Dyspepsia	7
29. Dysuria	1
30. Epilepsy	2
31. Gastroenteritis	7
32. Gastritis	43
33. General body pain	2

Conditions presented	Frequency
34. Headache	3
35. Heart related problems	6
36. Hemorrhoids	2
37. Hepatitis	1
38. Hepatoma	4
39. Hernia	2
40. Human Immunodeficiency Virus	1
41. Hyperglycaemia	1
42. Hypertension	143
43. Hypoglycaemia	3
44. Hypotension	1
45. Immunosuppression	2
46. Jaundice	1
47. Kaposi Sarcoma	7
48. Lactic acidosis	1
49. Leg numbness	1
50. Legs painful	1
51. Liver ascites	1
52. Liver cirrhosis	1
53. Lumbago	5
54. Left ventricular failure	1
55. Malaria	145
56. Malnutrition	2
57. Measles	19
58. Meningitis	10
59. Migraine	2
60. Musculoskeletal pain	31
61. Neck pain	3
62. Nephritis	4
63. Nephrotic syndrome	1
64. Neuroathic pains	1
65. Oesophageal	1
66. Oral Candidiasis	4
67. Otitis Media	1
68. Ovarian Mass	1
69. P. Neuropathy	5
70. Palpitation	1

71. Peripheral neuropathy	1
**Disease Presentation**	**Frequency**
72. Pelvic InflammatoryDisease	1
73. Pmd	22
74. Pneumonia	33
75. Post shingles	1
76. Psychological stress	2
77. Psychotic disorder	1
78. Pulmonary Tuberculosis	13
79. Peptic Ulcer disease	44
80. Renal failure	2
81. Right Heart Disease	3
82. Rheumatism	8
83. Respiratory Tract Infection	2
84. Severe Anaemia	3
85. Schistosoma haematobium infection	4
86. Sepsis	52
87. Severe Gastritis	2
88. Shigelliosis	1
89. Sickle cell disease	1
90. Sneezing	1
91. Shortness of breath	2
92. Sore throat	1
93. Stroke	2
94. Tuberculosis	6
95. Throat	1
96. Tonsilitis	4
97. TSS	1
98. Upper Respiratory Tract Infection	36
99. Urticaria	1
100. Urinary Tract Infection	13
101. Vaginal discharge	1
102. Worms	2
103. Unclear writing	7

Staff	Management	Patient (in-patients)	Affiliate
1 Clerk	Hospital director	2 from the 2A female ward	1 former hospital director
1 short stay ward nurse	MedDept HOD	2 from 2B male ward	1 laboratory staff
1 main ward nurse	MedDept matron	1 from 4B medical ward	1 pharmacy staff
1 student intern	MedDept registrar		1 former MedDept HOD (written interview)
1 clinician (non-respondent)	1 MedDept senior specialist		1 former MedDept HOD (non-respondent)

APPENDIX 3: CATEGORIES OF IN-DEPTH INTERVIEWEES
